# Value of Alvarado scoring system in diagnosis of acute appendicitis

**DOI:** 10.1016/j.amsu.2022.103642

**Published:** 2022-04-19

**Authors:** Mounir Bouali, Yassine El Berni, Aziz Moufakkir, Abdelilah El Bakouri, Khalid El Hattabi, Fatimazahra Bensardi, Abdelaziz Fadil

**Affiliations:** a Faculty of Medicine and Pharmacy, Hassan II University of Casablanca, Casablanca, Morocco; b Department of of Visceral Surgical Emergency, CHU Ibn Rochd, Casablanca, Morocco

**Keywords:** Appendicitis, Appendectomy, Alvarado score, Abdominal pain

## Abstract

**Introduction:**

and importance: Acute appendicitis is one of the most common causes of acute abdomen in surgical patients. The objectives of the study were to evaluate efficacy of Alvarado scoring system in preoperative diagnosis of acute appendicitis and correlating it with postoperative findings.

**Methods:**

The present study was a prospective study of 208 patients presenting with symptoms and signs of acute appendicitis to the emergency department during a period of 10 months. Patients who met the inclusion criteria were evaluated using Alvarado scoring system. The efficacy of Alvarado scoring system was assessed by calculating sensitivity, specificity, positive predictive value, negative predictive value and negative appendectomy rate.

**Results:**

Total 208 patients were included in the study, which included 142 males and 66 females, at score of 7 or more, appendicitis was confirmed in 187/190 patients, while at scores <7 appendicitis was confirmed in 10/18 patients. The sensitivity was 94.9%, the specificity was 72.7%, the positive predictive value was 98.4% and the negative predictive value was 44.4%. In the present study, negative appendectomy rate was 4.8%

**Conclusion:**

Clinical experience remains of major importance in diagnosing acute appendicitis. The Alvarado score is a simple, easy scoring system at both end of scale.

## Highlights

• Acute appendicitis is one of the most common causes of acute abdomen in surgical patients.
• Various scoring systems are used for decision making in appendicitis.
• Alvarado score remains the most popular scoring system.

## 1 Introduction

Acute appendicitis is one of the most common surgical emergencies [1]. The diagnosis of acute appendicitis is hampered by the absence of typical symptoms and of suggestive laboratory data in about 20–33% of the cases [2]. A quick and correct diagnosis of acute appendicitis with subsequent early appendectomy can avoid complications arising from perforation [3].

A high percentage of negative appendectomies (20%) was considered reasonable, based on the premise that delay would inevitably lead to perforation, increasing morbidity and even mortality [4]. The cost to both the patient and the healthcare system of negative appendectomies is considerable, and a complication rate of up to 6.1% following removal of normal appendices was reported [[5], [6], [7]]. These facts have made the need for a scoring test for the diagnosis, such as the Alvarado's Score, possible. It can contribute to the early detection of cases of acute appendicitis, reducing individual damage as well as social and material costs [2,8].

The present study aims to evaluate the efficacy of the Alvarado scoring system in the preoperative diagnosis of acute appendicitis and correlate it with postoperative findings.

## 2 Methods

We performed an analysis of prospectively collected data from 208 consecutive patients, above 15 years old, with suspected appendicitis, admitted to the Department of Visceral Surgical Emergency at the University Hospital Center Ibn Rochd, Casablanca, Morocco. The study ran from April 2020 to January 2021. All patients were given specific scores according to the variables of the Alvarado scoring system (Table 1) and then divided into 3 groups. Group 1: score 3 or less (unlikely acute appendicitis), group 2: score 4–6 (probably acute appendicitis), and group 3: score 7 or more (likely acute appendicitis).

**Table 1:** Alvarado score.

Variables	Clinical features	Score
Symptoms	Migratory RIF pain	1
	Anorexia	1
	Nausea and vomiting	1
Signs	Tenderness RIF	2
	Rebound tenderness	1
	Elevated temperature	1
Laboratory	Leucocytosis	2
	Shift to left	1
Total Score		10

The decision for appendectomy was taken by the qualified surgeon. Details of intraoperative findings were recorded and the definitive diagnosis was based on the histopathological assessment of the specimen. The efficacy of Alvarado scoring system was assessed by calculating sensitivity, specificity, positive predictive value, negative predictive value, and negative appendicectomy rate. this work has been registered with unique identification number 2464. SPSS statistical software was used to measure various score performance parameters. This work has been reported in line with the STROCSS criteria [9].

## 3 Results

**Fig. 1:**
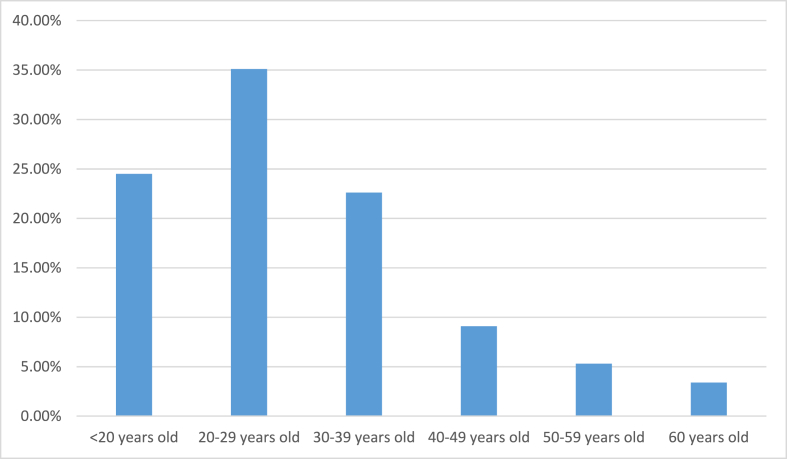
Age distribution.

In the study, a sample of 208 patients with suspected acute appendicitis was included. Of those patients, 142 (68.2%) were male and 66 (31.8%) were female. The patients' age range was from 15 to 74 years old, and the mean age was 29.5 years (Fig. 1). The most common symptom (apart from RIF pain, which is an inclusion criterion) was nausea and/or vomiting, which was present in 176 patients (90.4%), while the most common sign was RIF tenderness, with a frequency of 206 (99%) (Table 2).

**Table-2:** Individual features of Alvarado Score.

Clinical features	Numbers of patients	Percentage
Migratory RIF pain	65	20.3%
Anorexia	104	50%
Nausea and vomiting	176	90.4%
Tenderness RIF	206	99%
Rebound tenderness	82	39.4%
Elevated temperature	119	57.2%
Leucocytosis	193	92.8%
Shift to left	163	78.4%

Analysis of the subjects based on the Alvarado score indicates that 91% of the subjects exhibited a score of 7 or more, and 6% of the subjects had a score of between 4 and 6. Only 3% of the subjects had a score of between 1 and 3.

Findings (Table 3) reveal that the rate of negative appendectomy was very minimal, representing a percentage of 4.8%. On histopathological confirmation, 98.4% of patients had an acutely inflamed appendix with an Alvarado score ≥7, 75% with a score of between 4 and 6, and 16.7% with a score ≤3 (Table 4). The sensitivity, specificity, positive predictive value, negative predictive value, positive likelihood ratio, and negative likelihood ratio were 94.9%, 72.7%, 98.4%, 44.4%, 3.48, and 0.07, respectively.

**Table-3:** Appendectomy rate.

Variables	Numbers of patients	Percentage
Negative appendectomy	10	4.8%
Positive appendectomy	197	95.2%

**Table-4:** Correlation of alvarado score with histopathology.

Alvarado score	Results	Positive appendectomy	Negative appendectomy
7–10	190	187 (98.4%)	3 (1.6%)
4–6	12	9( 75%)	3 (25%)
1–3	6	1 (16.7%)	5 (83.3%)

## 4 Discussion

Various scoring systems were designed to decrease the negative appendectomy rate and increase the positive diagnostic rate of appendicitis [10]. Among them, a comprehensive scoring developed by “ALVARADO” in 1986 provides a practical diagnostic aid in interpreting the diagnosis of acute appendicitis [8]. The Alvarado's scoring system was introduced initially as an adjuvant to diagnose appendicitis to correct the previous false-positive diagnostic rate [11]. The Alvarado scoring system was simple, easily applicable, and useful in emergency surgical hospitals [12].

Epidemiological studies have shown that appendicitis is more common in the age 10–30 year group [13,14]. Our study also revealed a high incidence in the age <30 year group, in concordance with Limpawattanisiri C et al. [13]. In our study, males were more frequently affected than females, which is comparable to other studies [14,15].

In the present study, overall positive and negative appendectomy rates were 95.1% and 4.9%, respectively, which was comparable to other studies [[16], [17], [18]]. In our study, negative appendectomy at a score of >7 was 1.6%, which is comparable to Matija et al. study, which revealed no case of removal of the normal appendix at a score of >7 [19]. Thus, the Alvarado score showed a good correlation with the histopathological results: “the higher the score, the greater the incidence of histologically proven acute appendicitis.” Moreover, applying Alvarado's clinical scoring among the patients presenting with clinical manifestations of acute appendicitis in the emergency setup prevents false-negative operation [16].

In the present study, it was found that the application of Alvarado scoring provides 94.9% sensitivity, 72.7% specificity, 98.4% positive predictive value, and 44.4% negative predictive value in the diagnosis of acute appendicitis, taking histopathology as the gold standard. Our results match those of Kanumba et al. [20], who observed the sensitivity, specificity, positive predictive, negative predictive values, and accuracy of the Alvarado score to be 94.1%, 90.4%, 95.2%, and 88.4% respectively.

## 5 Conclusion

Clinical findings and experience remains of major importance in diagnosing acute appendicitis. Alvarado scoring system is useful tool in pre-operative diagnosis of acute appendicitis and can work effectively in routine practice.

## Provenance and peer review

Not commissioned, externally peer-reviewed.

## Sources of funding

None.

## Ethical approval

I declare on my honor that the ethical approval has been exempted by my establishment.

## Consent

Written informed consent for publication of their clinical details and/or clinical images was obtained from the patient. A copy of the written consent is available for review by the Editor-in-Chief of this journal on request.

## Author contribution

Mounir Bouali: writing the paper.

Yassine El Berni: writing the paper.

Aziz Moufakkir: writing the paper.

Abdelilah El Bakouri: study concept.

Khalid El Hattabi: study concept.

Fatimazahra Bensardi: study concept.

Abdelaziz Fadil: correction of the paper.

## Research registration

None.

## Guarantor

Yassine El Berni.

## Declaration of competing interest

The authors declare having no conflicts of interest for this article.
